# Recent Advances in Substrate-Controlled Asymmetric Cyclization for Natural Product Synthesis

**DOI:** 10.3390/molecules22071069

**Published:** 2017-06-27

**Authors:** Jeyun Jo, Seok-Ho Kim, Young Taek Han, Jae-Hwan Kwak, Hwayoung Yun

**Affiliations:** 1 College of Pharmacy, Pusan National University, Busan 46241, Korea; jju02160@gmail.com; 2 College of Pharmacy, Institute of Pharmaceutical Sciences, Cha University, Pocheon-si 11160, Korea; ksh3410@cha.ac.kr; 3 College of Pharmacy, Dankook University, Cheonan 31116, Korea; hanyt@dankook.ac.kr; 4 College of Pharmacy, Kyungsung University, Busan 48434, Korea; jhkwak@ks.ac.kr

**Keywords:** asymmetric cyclization, substrate-controlled manner, total synthesis, natural product

## Abstract

Asymmetric synthesis of naturally occurring diverse ring systems is an ongoing and challenging research topic. A large variety of remarkable reactions utilizing chiral substrates, auxiliaries, reagents, and catalysts have been intensively investigated. This review specifically describes recent advances in successful asymmetric cyclization reactions to generate cyclic architectures of various natural products in a substrate-controlled manner.

## 1. Introduction

Asymmetric construction of structurally diverse ring architectures has always been considered a formidable task in natural product synthesis. Various natural sources have provided an enormous number of enantiomerically enriched carbo- and heterocycles [1,2,3,4,5]. Their ring systems include monocycles, such as small-sized, medium-sized, and large-sized rings and polycycles, such as spiro-, fused-, bridged-, and ansa-rings. These intriguing structures have attracted considerable attention from the organic synthesis communities. Many synthetic chemists have explored fascinating tactics to construct chiral ring frameworks using chiral substrates, auxiliaries, reagents, and catalysts [6,7,8]. In particular the substrate-controlled cyclization strategy, which utilizes the nature of the built-in chiral environment in the starting material, is a very powerful method. In addition, this strategy is more environmentally and economically attractive than using chiral auxiliaries or catalysts [9,10].

The aim of this review is to highlight outstanding achievements in the construction of enantioenriched rings in the field of total synthesis from 2010 to April 2017. Selected original articles in this review contain various substrate-controlled cyclization strategies. The cyclization reactions are categorized by reaction type, such as anionic, cationic, transition metal-mediated, pericyclic, and radical reactions.

## 2. Anionic Cyclizations

**Scheme 1:**
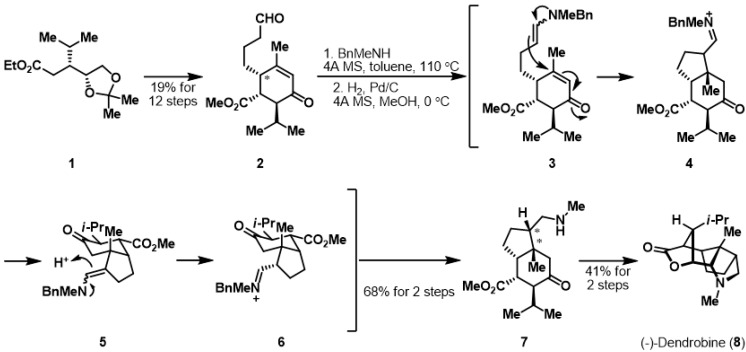
Total syntheses of (−)-dendrobine (**8**).

Recently, a wide range of natural product syntheses via anionic cyclization in a substrate-controlled manner have been reported. Carreira et al. accomplished the total synthesis of (−)-dendrobine (**8**) [11], which was isolated from the ornamental orchid *Dendrobium nobile* Lindl [12,13]. To construct the core of **8**, they utilized a special cascade sequence including enamine conjugate addition and stereoselective protonation as shown in Scheme 1. Highly advanced precursor **2** was readily prepared from ester **1** [14] in twelve steps. Treatment of **2** with *N*-methylbenzylamine followed by exposure to H_2_ and Pd/C led to bicycle **7** in 68% yield. High diastereoselectivity was obtained by a substrate-controlled cascade process involving the conjugate addition of enamine **3**, enamine formation by proton loss of **4** and the convex protonation of enamine **5**. This transformation allowed the asymmetric formations of the important C–C bond and the desired pendant amine.

**Scheme 2:**
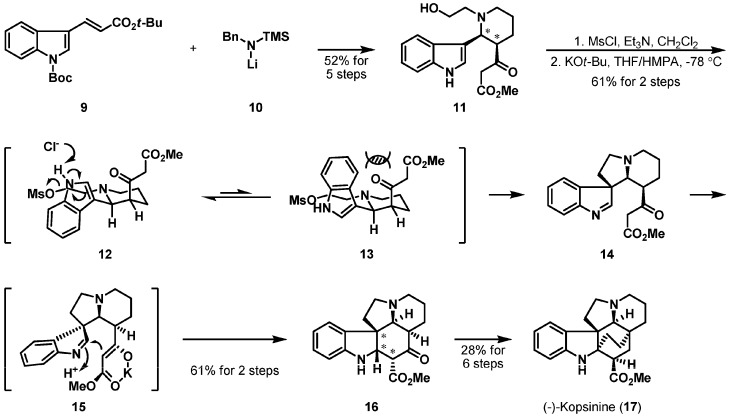
Total synthesis of (−)-kopsinine (**17**).

Another substrate-controlled asymmetric cyclization is summarized in Scheme 2 which depicts the total synthesis reported by Tomioka et al. [15] of (−)-kopsinine (**17**), a *Kopsia* alkaloid isolated from *Kopsia longiflora* Merr. in 1955 [16,17]. Key precursor **11** was smoothly formed via a high-yielding process including the asymmetric one-pot [N+2+3] cyclization of *tert*-butyl *N*-Boc-indole-3-propenoate (**9**) and lithium *N*-benzyltrimethylsilylamide (**10**). Having successfully inserted two stereocenters onto **11**, the three stereocenters of pentacyclic intermediate **16** were generated in a substrate-controlled manner. Mesylation of β-ketoester **11** and subsequent anionic cyclization of the resulting tetracycle **14** gave optically pure **16**, presumably through transition states **12** and **15**. The bridged ring of **17** was later introduced by Diels-Alder cyclization.

**Scheme 3:**
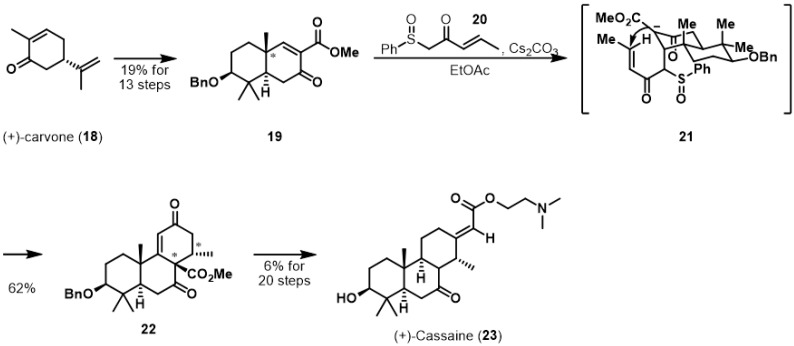
Total syntheses of (+)-cassaine (**23**).

An elegant anionic polycyclization strategy of Deslongchamps et al. led to the total synthesis of (+)-cassaine (**23**) as shown in Scheme 3 [18]. (+)-Cassaine was isolated from *Erythrophleum guineense* in 1935 and reported as a nonsteroidal Na^+^-K^+^-ATPase inhibitor [19]. (+)-Carvone (**18**) was selected as the starting building block to synthesize the pharmacologically interesting natural product. The authors successfully prepared cyclization precursor **19** based on their previous synthetic route [20]. With the asymmetric formation of the *trans*-decalin system of **19**, the desired anionic cyclization was performed using Nazarov reagent **20**. Thus, treatment with cesium carbonate in EtOAc gave rise to diastereomerically pure tricycle **22** via Michael adduct **21** in 62% yield. The newly created stereocenters originated from the α-face attack of **20** toward **19**, which was controlled by the steric repulsion of the angular methyl group in the ring junction of **19**. Synthesis of (+)-cassaine (**23**) was completed through multiple chemical reactions.

**Scheme 4:**
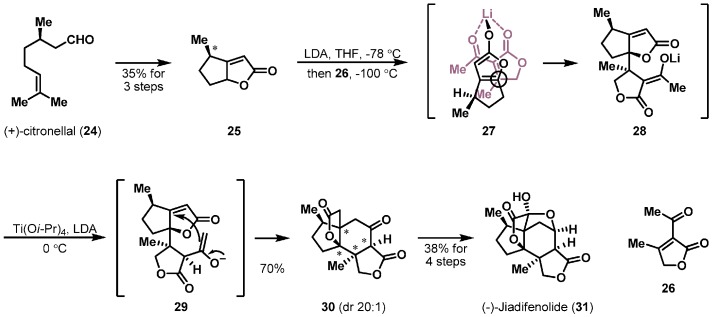
Total synthesis of (−)-jiadifenolide (**31**).

Stereoselective double Michael reactions have also been adopted for the construction of unique ring systems. Shenvi et al. completed the impressive synthesis of (−)-jiadifenolide (**31**) [21], which is one of the *Illicium* terpenes [22], as shown in Scheme 4. Key precursors butenolides **25** and **26** were quickly synthesized through three- and two-step routes, respectively. The first intermolecular Michael reaction of chiral butenolide **25** with achiral butenolide **26** in the presence of lithium diisopropylamide (LDA) provided stable intermediate **28**. The stereochemistry of the process derived from a chelated transition state **27** and the newly created stereocenters of **28** were controlled by the chiral methyl group of **25**. Subsequent exposure of the resulting **28** to titanium(IV) isopropoxide and additional LDA finally furnished ketolactone **30** via enolate **29** in 70% yield. The second intramolecular Michael reaction enabled the construction of the entire skeleton of **31** in a substrate-controlled manner.

## 3. Cationic Cyclizations

**Scheme 5:**
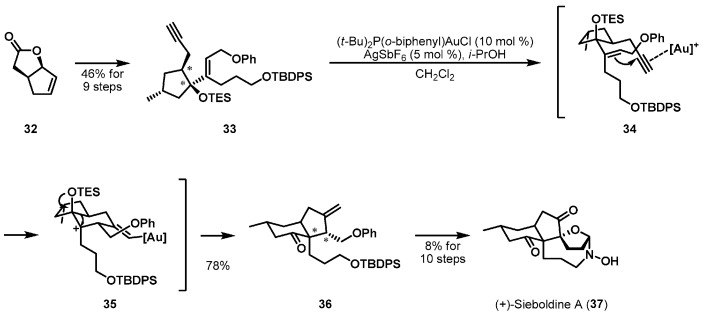
Total synthesis of (+)-sieboldine A (**37**).

Cation-induced cyclization has been a powerful tool for controlling the stereochemistry of various ring structures in natural product synthesis. The total synthesis of (+)-siebolidine A (**37**), which is an alkaloid of club moss *Lycopodium sieboldii* [23], was firstly reported by Overman et al. [24,25]. This landmark synthesis was accomplished with a pinacol-terminated cyclization cascade as depicted in Scheme 5. Cyclization precursor **33** was built from the readily available allylic lactone **32** [26,27] in 9 steps. Initially, an increased cationic environment of gold alkyne species **34** enabled 1,6-enyne cyclization. The subsequent pinacol shift of cyclized cationic intermediate **35** afforded the desired *cis*-hydrindanone **36** as a single stereoisomer. The stereochemistry of enyne **33** was efficiently reorganized in a substrate-controlled manner.

**Scheme 6:**
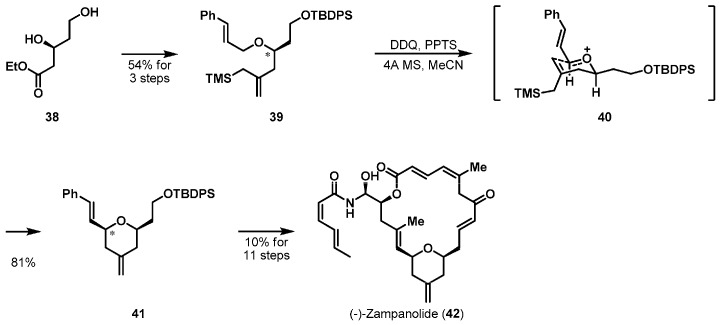
Total synthesis of (−)-zampanolide (**42**).

Another interesting example of substrate-controlled cyclization is the total synthesis of (−)-zampanolide (**42**) published by Ghosh et al. [28,29] (Scheme 6). (−)-Zampanolide was initially separated from the marine sponge *Fasciospongia rimosa* and exhibited a potent microtubule- stabilizing activity [30,31]. Synthesis of interesting macrolide **42** commenced from the known ester **38**, which was effectively prepared by the Noyori hydrogenation procedure [32]. Optically active starting material **38** was converted to allylsilane **39** in three steps. With the desired starting material **39** in hand, the authors carried out an oxidative Sakurai type cyclization using 2,3-dichloro-5,6-dicyano-1,4-benzoquinone (DDQ) with mild Brønsted acid pyridinium *p*-toluenesulfonate (PPTS). Cyclized product **41** was obtained diastereoselectively through the Zimmerman-Traxler transition state **40**. The *cis*-stereochemistry of tetrahydropyran ring **41** was caused by the equatorial orientations of all substituents in **40**. Having successfully assembled the core ring, macrocycle **42** was successfully constructed via cross and ring-closing metatheses.

**Scheme 7:**
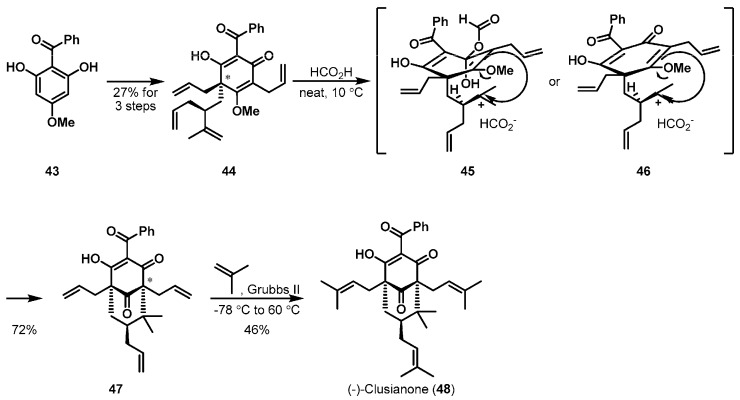
Total synthesis of (−)-clusianone (**48**).

Natural product clusianone is one of the polyprenylated polycyclic acylphloroglucinols (PPAPs) possessing a structurally unique bicyclo[3.3.1]nonane-2,4,9-trione core [33]. A biomimetic cationic cyclization was applied to construct the bicyclic template of (−)-clusianone (**48**) as shown in Scheme 7 [34]. Porco Jr. et al. were interested in the synthesis and structure-activity relationship of PPAP natural products and derivatives. Cyclization precursor **44** was accessible from starting material **43** via an unprecedented alkylative dearomatization strategy. Thus, when optically active **44** was subjected to neat formic acid, a tertiary carbocation was initially formed, which led to the intramolecular attack of the methyl enol ether to stereoselectively furnish the desired bicycle **47** in 72% yield. The authors proposed the transition states **45** and **46** due to the observation of formate adduct from ultrahigh performance liquid chromatography (UPLC) measurements. A final olefin metathesis produced (−)-clusianone (**48**).

**Scheme 8:**
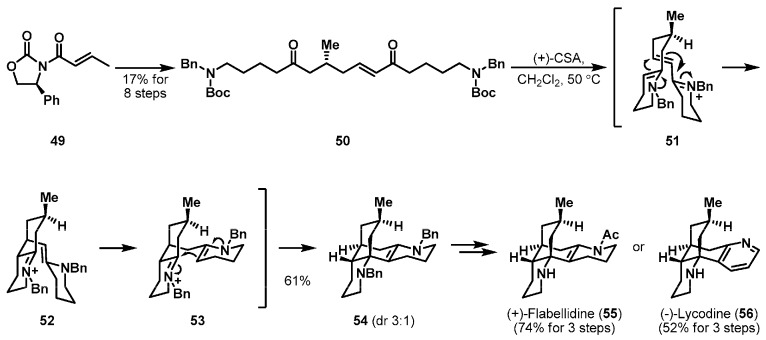
Total syntheses of (+)-flabellidine (**55**) and (−)-lycodine (**56**).

As depicted in Scheme 8, another biomimetic cationic cyclization study was performed by Takayama et al. [35]. *Lycopodium* alkaloids (+)-flabellidine (**55**) and (−)-lycodine (**56**) were reported from *Lycopodium complanatum* in 1942 and *Lycopodium annotinum* in 1958, respectively [36,37]. The methyl group of linear precursor **50** was diastereoselectively introduced by Hosomi-Sakurai allylation of commercially available crotonamide **49** [38]. With linear substrate **50** in hand, the authors examined the designed cationic cascade cyclization to form the tetracyclic backbone. Exposure of **50** in CH_2_Cl_2_ to an excess amount of (+)-camphorsulfonic acid (CSA) provided tetracycle **54** as the major product, presumably through conjugate addition of ene-iminium intermediate **51** and olefin migration of **52**, Mannich-like reaction of **53**. The diastereoselectivity was dominantly controlled by the stereocenter of the methyl group. After protecting group manipulation a minor diastereomer of the cationic cascade cyclization could be removed, and syntheses of (+)-flabellidine (**55**) and (−)-lycodine (**56**) were completed by a chemoselective acetylation and selective IBX oxidation, respectively.

**Scheme 9:**
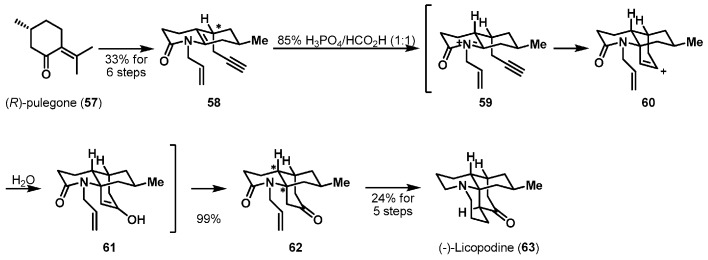
Total synthesis of (−)-lycopodine (**63**).

More recently, a protecting-group-free total synthesis of (−)-lycopodine (**63**) was described (Scheme 9). (−)-Lycopodine is one of the *Lycopodium* alkaloids and was originally isolated from *Lycopodium complanatum* in 1881 [39]. To construct its polycyclic architecture, She et al. employed an acid-promoted aza-Prins cyclization [40]. Cyclization substrate **58** was smoothly synthesized from (*R*)-pulegone (**57**) via Wade’s enone synthesis, a one-pot amidation, and cyclization [41]. Alkyne-enamide **58** was subjected to a phosphoric acid-promoted cyclization and the desired product **62** was obtained in almost quantitative yield. Initially, enamine **58** was protonated stereoselectively to furnish *N*-acyliminium **59**. Subsequent intramolecular 6-*exo*-trig cyclization provided unstable carbocation species **60,** which was quickly transformed to enol **61** via the capture of water. Further manipulation, including an intramolecular aldol cyclization, led to completion of the synthesis of (−)-licopodine (**63**).

## 4. Transition Metal-Mediated Cyclizations

**Scheme 10:**
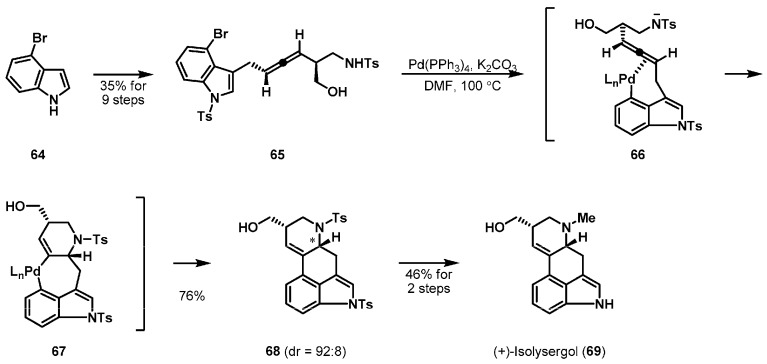
Total synthesis of (+)-isolysergol (**69**).

Transition metals have been used as powerful reagents for cyclization in the synthesis of complex natural products. The enantioselective total synthesis of (+)-isolysergol (**69**) [42], one of the biologically important ergot alkaloids [43], was completed by Fujii and Ohno et al. The authors examined a special palladium-catalyzed domino cyclization to build the ergot alkaloid backbone (Scheme 10). Allenic amide **65**, required for the cyclization, was prepared from commercially available 4-bromoindole (**64**). Successful substrate-controlled cyclization of allene **65** in the presence of 5 mol % of Pd(PPh_3_)_4_ and K_2_CO_3_ was achieved leading to the desired product **68** in 76% yield with high diastereoselectivity. After oxidative addition, aminopalladation of the indolylpalladium halide proceeded via the conformation **66** to provide alkenylpalladium(II) intermediate **67**. The stereochemistry of the ring junction in **68** ultimately derived from chiral allene **65**.

**Scheme 11:**
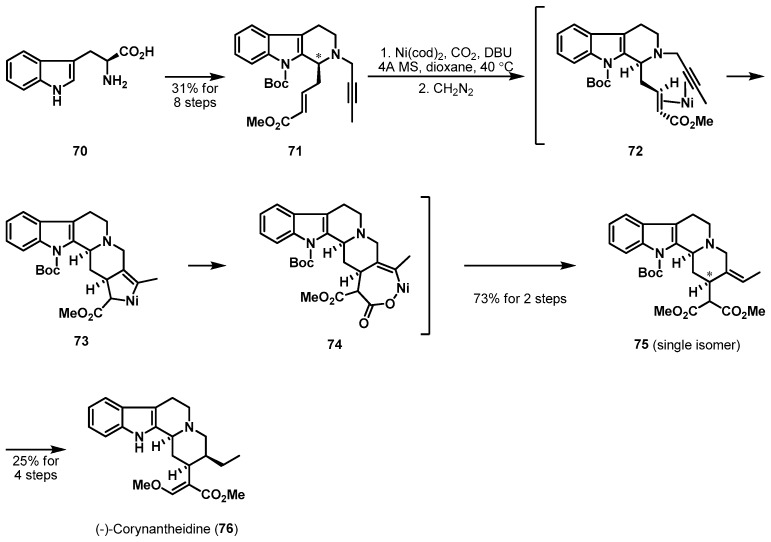
Total synthesis of (−)-corynantheidine (**76**).

The nickel-mediated carboxylative cyclization of enyne precursor is another example of a transition-metal mediated cyclization as depicted in Scheme 11. Sato et al. described the total synthesis of indole alkaloid (−)-corynantheidine (**76**) [44], which was first reported in 1944 from the African plant *Pseudocinchona africana* [45]. Desired enyne **71** was readily accessible from l-tryptophan (**70**) in an optically active form through a sequence of usual transformations involving a *cis*-selective Pictet-Spengler reaction [46]. Upon treatment of precursor **71** with a stoichiometric amount of Ni(cod)_2_ and 1,8-diazabicyclo[5.4.0]undec-7-ene (DBU) under an atmosphere of gaseous CO_2_, the crude materials were obtained. Hydrolysis and methylation of the crude nickelacycle **73** stereoselectively led to the desired tetracycle **75** in 73% yield. The fourth ring was formed through oxidative cycloaddition of **71** and subsequent insertion of CO_2_ between the C_sp3_–nickel bond in **73**. The new stereogenic center of the ring junction in **75** was created by an asymmetric formation of the nickelacycle in a substrate-controlled manner.

**Scheme 12:**
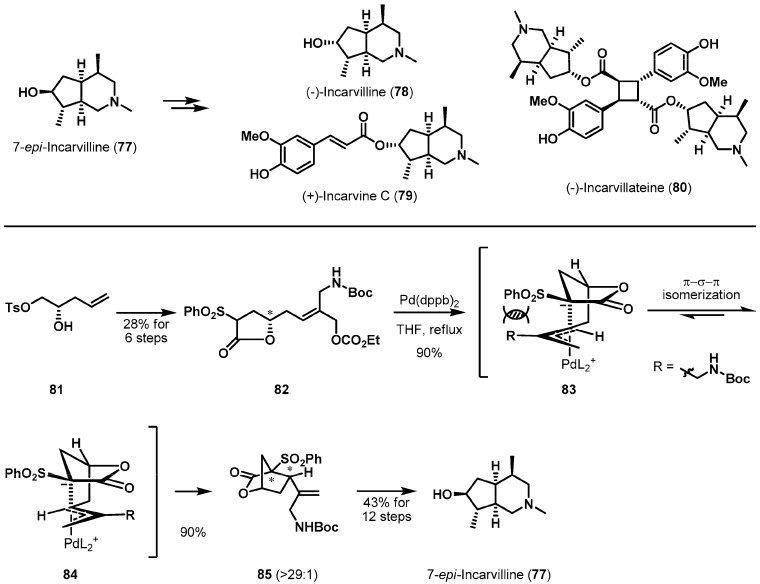
Synthesis of 7-*epi*-incarvilline (**77**).

The asymmetric synthesis of incarvillea alkaloids was studied by using palladium(0)-catalyzed cyclization. Suh et al. completed an enantioselective synthesis of 7-*epi*-incarvilline (**77**) [47], which is an advanced architecture for the formal syntheses of (−)-incarvilline (**78**), (+)-incarvine C (**79**), and (−)-incarvillateine (**80**) (Scheme 12). Structurally, they consist of a common bicyclic piperidine skeleton including five contiguous stereocenters [48,49,50]. The authors focused on the diastereoselective construction of key intermediate **85**, a highly functionalized bicyclic lactone containing three stereocenters. The desired precursor **82** of the reaction was easily obtained from the known chiral tosylate **81 [51]**. Exposure of **82** in THF to Pd(dppb)_2_ resulted in the desired bicyclic lactone **85** in 90% yield and with excellent diastereoselectivity (>29:1). The two transition states, **83** and **84**, were controlled by the built-in chirality of lactone **82** and the high diastereoselectivity likely occurred due to the steric repulsion between the benzenesulfonyl group and the R substituent in Pd–π-allyl complex of **83**. With the bicyclic lactone **85** in hand, 7-*epi*-incarvilline (**77**) was synthesized using further manipulations such as a substrate-controlled catalytic hydrogenation and a 1,4-addition.

**Scheme 13:**
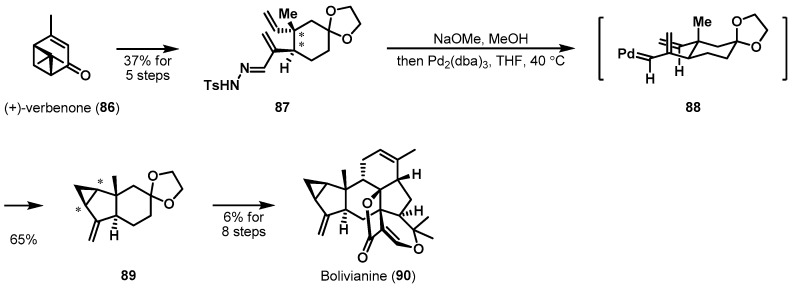
Total synthesis of bolivianine (**90**).

Metal-catalyzed carbenoid transfer is also a useful method for asymmetric cyclizations. Bolivianine was isolated from *Hedyosmum angustifolium* (Chloranthaceae) in 2007 [52]. The sesterterpenoid natural product consists of a highly complex heptacyclic skeleton and nine stereocenters. Liu et al. reported a bioinspired total synthesis of bolivianine (**90**), which is summarized in Scheme 13. [53,54]. Precursor tosylhydrazone **87** was well designed for the formation of the chiral cyclopropyl moiety in **89** and produced from commercially available (+)-verbenone (**86**). The programmed intramolecular cyclopropanation of **87** with a palladium catalyst and a sodium salt afforded the desired product **89** as the sole isolable diastereomer in 65% yield. The stereochemistry of the chiral cyclopropane was substrate-controlled via allylic metal carbene species **88**, which its conformation was caused by an equatorial positioning of two alkyl chains in chair-like transition state.

## 5. Pericyclic Reactions

**Scheme 14:**
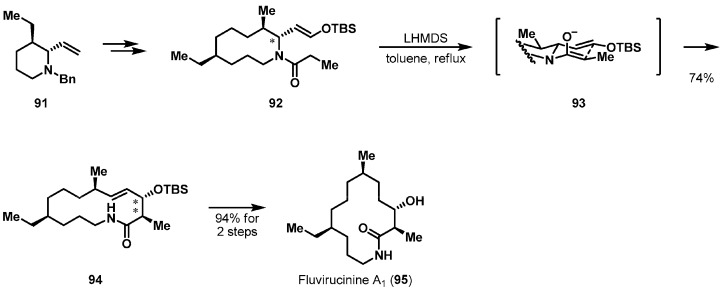
Total synthesis of fluvirucinine A_1_ (**95**).

**Scheme 15:**
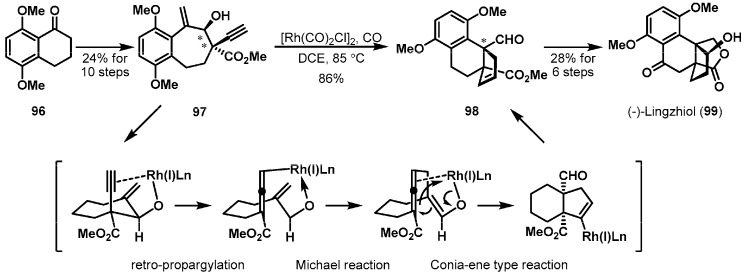
Total synthesis of (−)-lingzhiol (**99**).

Diverse types of pericyclic reactions have been used as an attractive tactic for substrate-controlled asymmetric cyclizations. Structurally interesting fluvirucins were isolated from actinomycetes by scientists from Bristol-Myers Squibb [55,56,57,58] and Schering-Plough [59,60] independently. Suh et al. employed a stereoselective aza-Claisen rearrangement-promoted ring expansion in the total synthesis of antibiotic macrolactam fluvirucinine A_1_ (**95**), an aglycon of fluvirucine A_1_ (Scheme 14) [61]. The key precursor, 10-membered α-alkoxyvinyl acylazacycle **92**, was obtained from piperidine **91** by a diastereoselective α-alkylation of an iminium ion generated from an *N*,*O*-acetal TMS ether [62]. With the functionalized precursor **92** in hand, the projected aza-Claisen reorganization was executed in the presence of lithium *bis*(trimethylsilyl)amide (LHMDS) in refluxing toluene, to affording 14-membered macrolactam **94**. The origin of the stereoselectivity could be explained based on a selective (*Z*)-lactam enolate formation and an equatorial positioning of the alkoxyvinyl substituent in chair-like transition state **93**.

(−)-Lingzhiol and its enantiomer were separated from *Ganoderma lucidum* in 2013 and found to inhibit selectively p-Smad3 [63]. The architecture of lingzhiol is composed of a carbocyclo[4.3.0]nonane skeleton and two quaternary bridgehead carbons (Scheme 15). Long et al. achieved the total synthesis of (−)-lingzhiol (**99**) via a rhodium-catalyzed intramolecular [3 + 2] cycloaddition [64]. Important precursor **97** was easily generated from 5,8-dimethoxy-3,4-dihydronaphthalen-1(2*H*)-one (**96**) in 10 steps by a ring expansion reaction employing Koser’s reagent [65] and an alkynylation using Waser’s reagent [66]. Treatment of compound **97** with the rhodium catalyst in the presence of CO in DCE gave rise to tricycle **98** in a diastereoselective manner. The catalytic cycle underwent a retro-propargylation, Michael reaction, Conia-ene type reaction, and protonolysis. The stereochemistry of the product was transferred stereospecifically from the chirality of the substrate. Further six steps completed the synthesis of (−)-lingzhiol (**99**).

**Scheme 16:**
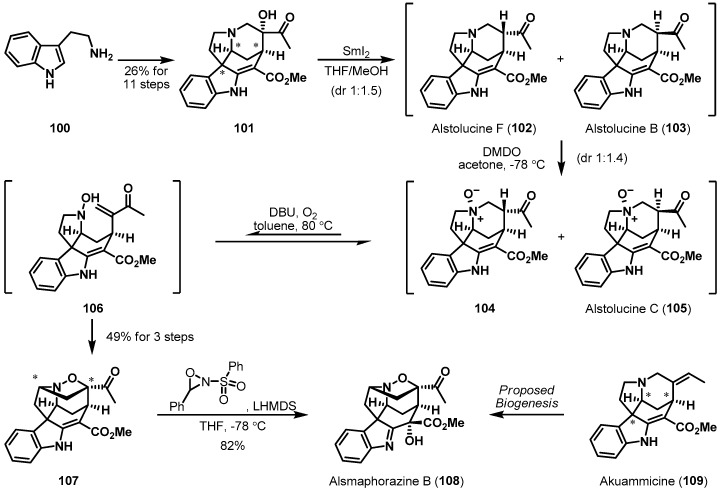
Total synthesis of alsmaphorazine B (**108**).

Vanderwal et al. recently described the total synthesis of alsmaphorazine B (**108**) by an intramolecular nitrone/alkene dipolar cycloaddition (Scheme 16) [67]. Alsmaphorazine B possesses a highly dense, oxidized, and cage-like hexacyclic structure with a rare *endo* N–O bond [68]. The authors analyzed the structure of **108** and then proposed an alternative biogenetic hypothesis of multiple oxidations from akuammicine (**109**). Akuammicine-derived pivotal substrate **101** was prepared from tryptamine (**100**) by applying a scalable process involving a Diels-Alder reaction and a Heck reaction. Firstly, deoxygenation of α-ketol **101** with samarium iodide furnished two diastereomers, **102** and **103**, in 1:1.5 ratio. Subsequent DMDO oxidation of the mixture of tertiary amines **102** and **103** readily led to the corresponding *N*-oxides, **104** and **105**. Then, treatment of the obtained *N*-oxide mixture, **104** and **105**, with DBU liberated a hydroxylamine and an enone. Finally, the desired cycloadduct **107** was spontaneously produced in 49% yield for 3 steps. This surprising sequence proceeded stereoselectively in a substrate-controlled manner.

**Scheme 17:**
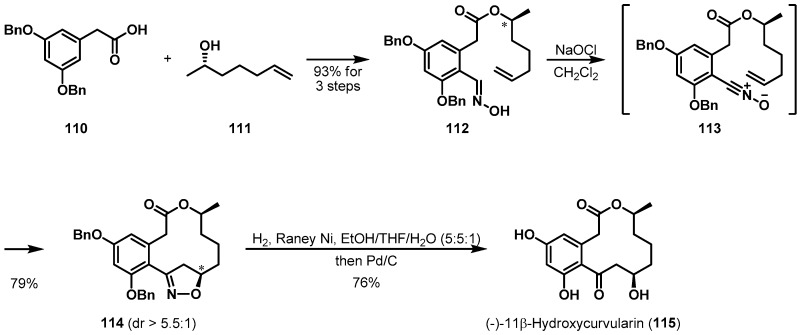
Total synthesis of (−)-11β-hydroxycurvularin (**115**).

Intramolecular nitrile oxide-olefin cycloaddition (INOC) was utilized to form the macrocyclic moiety of (−)-11β-hydroxycurvularin (**115**) (Scheme 17). This polyketide was isolated from *Alternaria tomato* and contains a 3,5-dihydroxyphenylacetic acid skeleton [69]. Lee et al. described a remote stereoinductive fashion of the key cyclization to synthesize the natural product [70]. Oxime **112**, the substrate of nitrile oxide **113**, was efficiently prepared from commercially available **110** by esterification, formylation, and oxime formation. Then oxime **112** was subjected to an intramolecular nitrile oxide cycloaddition reaction. It is well known that isolated alkene group has decreased reactivity compared to enone group and that regio- and stereoselectivity can be problematic in large ring formation with remote stereocenter [71]. Unexpectedly, the desired isomer **114** was produced in 79% yield with good diastereoselectivity. This reaction was an example of unprecedented remote stereoinduction and concomitant macrocyclization.

**Scheme 18:**
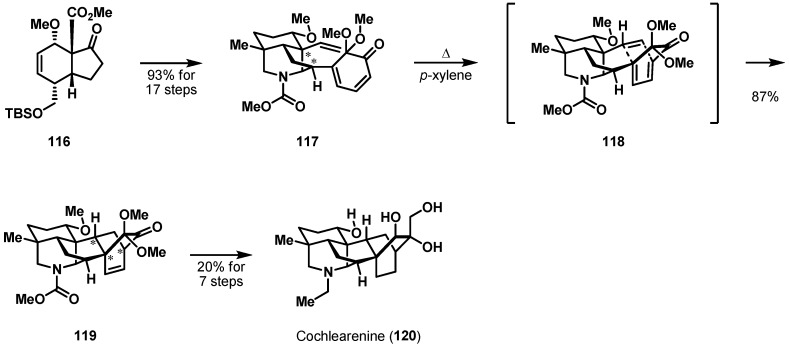
Total synthesis of cochlearenine (**120**).

More recently, unified syntheses of denudatine-type diterpenoid alkaloids were disclosed by Sarpong et al. [72]. Among these alkaloids, cochlearenine (**120**) consists of a highly complex bicyclo[2.2.2] structural architecture and exhibits a bradycardic activity in guinea pig atria [73,74]. Synthesis of cyclization precursor **117** began with the known bicycle **116**, which was effectively prepared using Diels-Alder cycloaddition [75] (Scheme 18). Subjecting a solution of dienone **117** in *p*-xylene to heat resulted in the exclusive formation of hexacycle **119**, which contains the whole ring backbone of **120**. The excellent diastereoselectivity was determined by a substrate-controlled transition state. Total synthesis of cochlearenine (**120**) was completed in seven steps from hexacycle **119**.

## 6. Radical Cyclizations

**Scheme 19:**
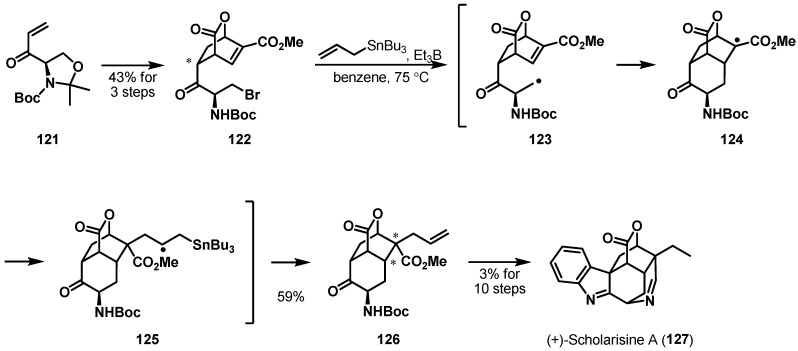
Total synthesis of (+)-scholarisine (**127**).

Radical cyclizations have served as a useful strategy to create various ring systems. As shown in Scheme 19, a total synthesis of the akuammiline alkaloid (+)-scholarisine A (**127**) was completed by Snyder et al. [76]. The structurally unique alkaloid, which was isolated from *Alstonia scholaris*, contains an indolenine fused to a strained carbocyclic cage and several tertiary and quaternary stereogenic centers [77]. To access the cage architecture of **126**, a tandem 6-*exo*-trig radical cyclization/Keck allylation was explored. The key precursor **122** of the tandem reaction was efficiently prepared in three steps from acrylate **121** [78]. Substrate **122** was smoothly cyclized using allyltributylstannane and the radical initiator Et_3_B [79] in benzene at 75 °C, providing the desired **126** in 59% yield as a single diastereomer. The transformation likely progressed in the mechanistic fashion shown in Scheme 19 through the initial radical formation of **123**, 6-*exo*-trig cyclization onto the Michael accepter of **124**, and allylation of the resulting **125**. The newly incorporated stereogenic centers were influenced by the asymmetric geometry of bicyclic lactone **122**.

**Scheme 20:**
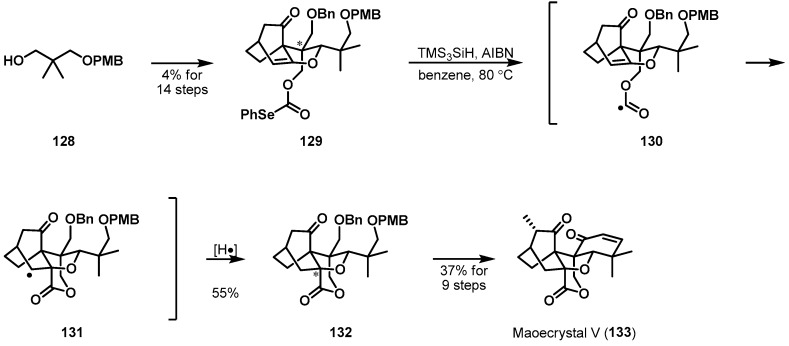
Total synthesis of maoecrystal V (**133**).

Another remarkable radical cyclization was reported by Zakarian et al. (Scheme 20) [80]. Maoecrystal V (**133**), a cytotoxic diterpenoid, was discovered in 2004 from *Isodon eriocalyx*, a Chinese medicinal herb [81]. Structurally, three contiguous quaternary stereocenters are compactly arranged on a pentacyclic framework; the complex molecular architecture provides an intriguing synthetic challenge. Key precursor **129**, having two quaternary stereocenters, was synthesized from alcohol **128** through a C–H-insertion and a [4 + 2] cycloaddition. With phenylselenocarbonate **129** in hand, a substrate-controlled radical cyclization was explored. To a solution of substrate **129** in benzene at 80 °C was slowly added a mixture of TMS_3_SiH, as a less efficient hydrogen atom donor reagent, and AIBN, resulting in the formation of lactone **132** (55% yield). The asymmetric cyclization successfully generated the third quaternary stereocenter of **132** through the initially generated formyl radical **130**.

**Scheme 21:**
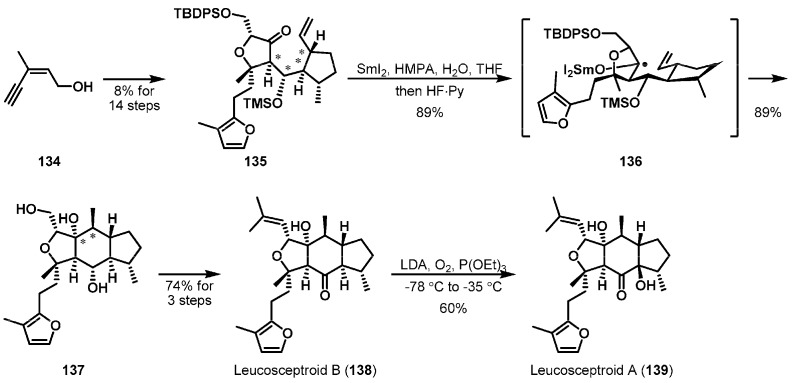
Total syntheses of leucosceptroids A (**139**) and B (**138**).

Ma et al. described the total syntheses of leucosceptroids A (**139**) and B (**138**) by applying a SmI_2_-mediated intramolecular ketyl-olefin radical cyclization (Scheme 21) [82]. These two sesterterpenoids were separated from glandilar trichomes of *Leucosceptrum canum* [83]. These intriguing compounds contain a common tricyclic hydrindane ring skeleton and eight contiguous stereogenic centers. Advanced intermediate **135** was smoothly accessed from commercially available enynol **134**. Exposure of **135** to samarium(II) iodide gave rise to fused tricycle **137** through ketyl radical species **136** in a substrate-controlled manner. The α-OTMS unit of **136** played a critical role to promote selective 6-*exo*-cyclization, which competed with 7-*endo*-cyclization, by blocking the chelation of the free hydroxy group to SmI_2_, and high diastereoselectivity by decreasing the steric repulsion with the olefin. Further transformations efficiently led to completion of the syntheses of leucosceptroids A (**139**) and B (**138**).

**Scheme 22:**
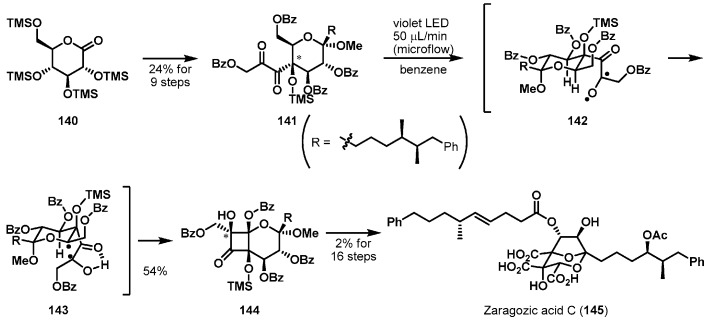
Total synthesis of zaragozic acid C (**145**).

More recently, a remarkable photochemical C–H acylation was utilized to cyclize a complicated ring system (Scheme 22). Inoue et al. presented a total synthesis of zaragozic acid C (**145**) [84], a potent inhibitor of mammalian squalene synthase [85]. Natural acid **145** is characterized by a dioxabicyclo[3.2.1]octane architecture with an array of six stereocenters. Pivotal radical cyclization precursor **141** was produced from commercially available gluconolactone derivative **140**. Irradiation of highly oxygenated substrate **141** with violet LED light excited the 1,2-diketone moiety and the resulting 1,2-biradical **142** spontaneously generated 1,4-biradical **143** via a hydrogen atom abstraction of the proximal ethereal C–H bond. The facile C–C bond formation of the 1,4-biradical **143** stereoselectively afforded the desired bicycle **144** (54% yield) by avoiding steric repulsions between the bulky substituents. Careful functional group transformations provide final product **145**.

## 7. Conclusions

Substrate-controlled asymmetric cyclizations provide organic chemists with a powerful tool for the construction of optically active ring frameworks of natural products. In this review, a remarkable variety of cyclizations were classified by reaction type, such as anionic, cationic, transition metal-mediated, pericyclic, and radical reaction and discussed in detail. Various ring systems of natural products and their newly generated stereocenters were successfully established and defined. To conclude, asymmetric cyclization induced by the chiral nature of the substrate is in continuous development for the synthesis of natural products and related compounds.

## Abbreviations

The following abbreviations are used in this manuscript:

Ac	Acetyl
AIBN	Azobisisobutyronitrile
Bn	Benzyl
Boc	*t*-Butoxycarbonyl
Bu	Butyl
Bz	Benzoyl
cod	1,5-Cyclooctadiene
CSA	Camphorsulfonic acid
dba	Dibenzylideneacetone
DBU	1,8-Diazabicyclo[5.4.0]undec-7-ene
DCE	1,2-Dichloroethane
DDQ	2,3-Dichloro-5,6-dicyano-1,4-benzoquinone
DMDO	Dimethyldioxirane
DMF	*N,N*-Dimethylformamide
dppb	1,4-Bis(diphenylphosphino)butane
Et	Ethyl
HMPA	Hexamethylphosphoramide
IBX	*o*-Iodoxybenzoic acid
LDA	Lithium diisopropylamide
LED	Light emitting diode
LHMDS	Lithium *bis*(trimethylsilyl)amide
Me	Methyl
MS	Molecular sieves
Ms	Mesyl
Ph	Phenyl
PMB	4-Methoxybenzyl
PPTS	Pyridinium *p*-toluenesulfonate
Pr	Propyl
Py	Pyridine
TBDPS	*t*-Butyldiphenylsilyl
TBS	*t*-Butyldimethylsilyl
TES	Triethylsilyl
THF	Tetrahydrofuran
TMS	Trimethylsilyl
Ts	*p*-Toluenesulfonyl
